# Advances in the Neurocognition of Music and Language

**DOI:** 10.3390/brainsci10080509

**Published:** 2020-08-02

**Authors:** Daniela Sammler, Stefan Elmer

**Affiliations:** 1 Otto Hahn Group Neural Bases of Intonation in Speech and Music, Max Planck Institute for Human Cognitive and Brain Sciences, 04103 Leipzig, Germany; 2 Auditory Research Group Zurich (ARGZ), Division Neuropsychology, Institute of Psychology, University of Zurich, 8050 Zurich, Switzerland

**Keywords:** music, language, brain, rhythm, prosody, musical training, dyslexia, reading, oscillations, statistical learning, cognitive control

## Abstract

Neurocomparative music and language research has seen major advances over the past two decades. The goal of this Special Issue “Advances in the Neurocognition of Music and Language” was to showcase the multiple neural analogies between musical and linguistic information processing, their entwined organization in human perception and cognition and to infer the applicability of the combined knowledge in pedagogy and therapy. Here, we summarize the main insights provided by the contributions and integrate them into current frameworks of rhythm processing, neuronal entrainment, predictive coding and cognitive control.

The scholarly fascination for the relationships between music and language (M&L) is as old as antiquity. To this day, continuous methodological progress and, in part, radical conceptual shifts paved the way for new directions of research. In the 1990s, technological revolutions in neuroimaging revealed partial neural overlap between the two domains [1], despite dissociable clinical deficits in M&L [2]. Together with known benefits of music for speech and language functions [3] this nurtured the idea that—once we understand what holds M&L together at their biological core—music interventions could constitute a bridge to prevent, alleviate, or even reverse speech and language disorders [4,5].

This Special Issue took stock of recent advances in the neurocognition of M&L to examine the current status of this vein of research. Sixteen research papers and reviews from 48 experts in linguistics, musicology, cognitive neuroscience, biological psychology and educational sciences demonstrate that research has been active on all fronts. As we will see, the studies follow two burgeoning trends in M&L research: First, they focused on common auditory processing of temporal regularities [6,7,8,9] that are thought to promote higher-level linguistic functions [8,10,11,12,13,14], possibly via mechanisms of neuronal entrainment [15]. Second, they explored top-down modulations of common auditory processes [16,17,18] by domain-general cognitive [19,20] and motor functions in both perception and production [21]. These topics were addressed using a broad toolkit of well-designed behavioral and computational approaches combined with functional magnetic resonance imaging (fMRI), near-infrared spectroscopy (NIRS) or electroencephalography (EEG) in different cohorts of participants.

The starting point for most of the included studies was that speech and music have similar acoustic [9,18] and structural features [6,7,8,13,15,16,17,19]. As argued in the review article of Reybrouck and Podlipniak [9], some of these sound features and their common preconceptual affective meanings may even reflect joint evolutionary roots of M&L that still prevail today, for example, in musical expressivity and speech prosody. Notably, a feature that was particularly central to half of all contributions is the *temporal* structure of M&L, i.e., the patterning of strong and weak syllables or beats that make up rhythm, meter and prosodic stress [6,7,8,10,11,12,13,15].

The rhythmic patterning of both speech and music has been proposed to draw on domain-general abilities which are required to perceive and process temporal features of sound [22,23]. Accordingly, three studies present data in line with common rhythm processing resources in M&L. First, Lagrois et al. [6] found that individuals with beat finding deficits in music—so called “beat-deaf” individuals—also show deficits in synchronizing their taps with speech rhythm, and more generally, in regular tapping without external rhythms. The authors argue that this pattern of deficits may arise from a basic deficiency in timekeeping mechanisms that affect rhythm perception across domains. Second, Boll-Avetisyan et al. [7] used multiple regression analyses and found that musical rhythm perception abilities predicted rhythmic grouping preferences in speech in adults with and without dyslexia. Similarly, in an EEG study, Fotidzis et al. [8] found that musical rhythmic skills predicted children’s neural sensitivity to mismatches between the speech rhythm of a written word and an auditory rhythm. Interestingly, both studies further report connections between rhythm perception in music and reading skills. Hence, these findings not only speak for a common cross-domain basis of rhythmic processes in M&L but also suggest that deficient or enhanced rhythmic abilities may have an impact on higher-level language functions.

Potential downstream effects of general rhythmic processing skills on higher-order linguistic abilities are currently being extensively investigated, particularly in the context of first language acquisition (for a recent review, see [24]). Accordingly, several studies in this Special Issue probe whether the acoustic properties of speech rhythm can serve as scaffolding for the acquisition of stable phonological representations [12], for the segmentation of words from continuous speech and the construction of lexical representations [13], for the recognition of syntactic units in sentences [10] and for reading [7,8,11]. For example, Richards and Goswami [10] explain that prosody, particularly the hierarchical structuring of stressed and unstressed syllables, provides reliable cues to the syntactic structure of speech [25] and can hence facilitate learning of syntactic language organization [26]. Early perturbations at this rhythm-syntax interface may, in turn, hinder normal language acquisition, such as in developmental language disorders (DLD). The authors found that children with DLD indeed had difficulties in noticing conflicting alignments between prosodic and syntactic boundaries in rhythmic children’s stories, and that these deficits coincided with enhanced perceptual thresholds for acoustic cues to prosodic stress. With these data at hand, Richards and Goswami support the assertion that basic processing of rhythmic-prosodic cues may be a key foundation onto which higher aspects of language are scaffolded during development.

In a similar vein, rhythmic-prosodic sensitivity has been proposed as fundamental stepping stone into literacy [27,28,29] as well as implicit driver for skilled reading [30]. Breen et al. [11] and Fotidzis et al. [8] present converging EEG evidence for implicit rhythmic processing in silent reading of words in literate adults and children. In particular, they both found a robust fronto-central negativity in response to stress patterns in written words that mismatched the rhythm of silently read limericks [11] or auditory click trains [8]. These results suggest that rhythmic context—no matter whether implicit in written text or explicit in sound—can induce expectations of prosodic word stress that facilitate visual word recognition and reading speed.

Current neurophysiological models assume that speech and music processing as well as the catalytic role of rhythm in language development are based on the synchronization of internal neuronal oscillations with temporally regular stimuli [27,31,32,33]. The review article by Myers et al. [15] summarizes the current state of knowledge about neuronal entrainment to the speech envelope reflecting quasi-regular amplitude fluctuations over time. This neural tracking occurs simultaneously at multiple time scales corresponding to the rates of phonemes, syllables and phrases [34,35]. In this context, Myers and colleagues argue that the slowest rate—corresponding to prosodic stress and rhythmic pacing in the delta range (~2Hz)—constitutes a particularly strong source of neuronal entrainment which is crucial for normal language development. Correspondingly, atypical entrainment to rhythmic prosodic cues due to deficits in fine-grained auditory perception may constitute a risk for the development of speech and language disorders such as DLD and developmental dyslexia (DD) [24,36].

If rhythmic processing disabilities are indeed the basis of speech and language disorders, then useful avenues for prevention and intervention could lie in (i) increasing the regularity of stimuli, or (ii) strengthening individual rhythmic abilities with the aim at improving neuronal entrainment [37,38,39]. Several studies in this Special Issue deal directly or indirectly with these ideas, either by exploring processing benefits of rhythmically highly regular stimuli such as songs [13,14] or poems [10,11], or by discussing potential protective or curative effects of music-based rhythm training on language skills [7,8,10,12,15,16]. Even though the results are promising, they also raise a number of questions. For example, using EEG Snijders et al. [13] found that 10-month-old infants were able to segment words in natural children’s songs. However, they did equally well in infant-directed speech. Similarly, Rossi et al. [14] found no differences between speech and songs in a combined EEG-NIRS study on semantic processing in healthy adults. Taken together, these data suggest that the presentation of verbal material as song may not be sufficient to enhance vocabulary learning or language comprehension in healthy individuals (but see [40]). The longitudinal study of Frey et al. [12] zoomed in on training effects. Using EEG, the authors demonstrate that 6 months of music but not painting training positively influenced the pre-attentive processing of voice onset time in speech in children with DD. However, no effects were found in behavioral measures of phonological processing or reading ability. This raises the questions of how much training is required and which aspects the training should include to translate to behavior, both inside and outside the laboratory setting. Clearly, the identification of optimal interventions is a joint mission for future research that goes hand in hand with the development of solid conceptual [41,42] and neurophysiological frameworks [27] to identify the key variables underlying the amelioration of speech and language processing through rhythm and music [43,44,45,46].

The studies of this Special Issue introduced so far primarily focused on links between M&L that are bottom-up driven by shared acoustic features between the two domains. The remaining articles took a different approach and examined domain-general top-down modulations of M&L from both the perspectives of perception and production. Four articles illustrate the continuous interaction between bottom-up and top-down processes. In line with significant trends in predictive coding [47,48], Daikoku [16] reviews the conceptual, computational, experimental and neural similarities of statistical learning in M&L acquisition and perception with links to rehabilitation. Bidirectional interactions between perceptual (bottom-up) and predictive (top-down) processes are a core feature in the framework of statistical learning. Experimental evidence for the top-down adjustment of M&L perception is provided by the behavioral modelling study of Silva et al. [17] who found that listeners placed break patterns in ambiguous speech-song stimuli differently depending on whether they believed they were listening to speech prosody or contemporary music. Similarly, the fMRI study of Tsai and Li [18] found that the strength with which an ambiguous stimulus was perceived as song rather than speech depended not only on the acoustics of the stimulus itself, but also on the sound category of the preceding stimulus. Finally, Mathias et al. [21] show with EEG that pianists gradually anticipated the sounds of their actions during music production, similar to mechanisms of auditory feedback control during speech production [49,50]. Taken together, these studies suggest that the listening context, one’s own motor plans as well as statistical and domain-specific expectations may influence the top-down anticipation and perception of acoustic features in speech and music.

Finally, the last two articles focus on the relevance of domain-general cognitive functions for M&L interactions. Lee et al. [19] argues that well-known syntax interference effects between M&L [51,52] may emerge from shared domain-general attentional resources. Accordingly, they show that the top-down allocation of attention similarly modulated EEG markers of syntax processing in M&L, particularly at late processing stages associated with cognitive reanalysis and integration. Otherwise, Christiner and Reiterer [20] found that links between musical aptitude and phonetic language abilities in pre-school children (i.e., imitation of foreign speech) were mediated by domain-general working memory resources. While none of these studies denies auditory-perceptual connections between M&L, they remind us that what we have seen so far is perhaps only the tip of the iceberg, with more complex entwinements still to be discovered.

To sum up, this Special Issue indicates that questions have shifted from mapping to mechanisms. Initial descriptions of M&L analogies have turned into a determined search for explanations of M&L links in human neurophysiology, general perceptual principles and cognitive computations. Accordingly, the obvious next questions are of a mechanistic nature: Can musical training enhance the neuronal entrainment to speech (and vice versa)? How exactly does entrainment promote higher-order linguistic functions? How can working memory and attention be included in the equation? These are only a few questions, but we are confident that the joint efforts of this multidisciplinary field of research will be rewarded by a better understanding of the M&L interface and the necessary tools to optimize interventions for music- and language-related dysfunctions.
